# Traditional Chinese medicine health literacy and depressive symptoms in postpartum women: the sequential mediating effects of social support and self-efficacy

**DOI:** 10.3389/fpubh.2026.1891379

**Published:** 2026-09-16

**Authors:** Dian Liu, Xiaomei Xu, Xiangui Lü

**Affiliations:** 1 School of Basic Medical Sciences & School of Nursing, Chengdu University, Chengdu, China; 2 Clinical Medical College & Affiliated Hospital of Chengdu University, Chengdu, China

**Keywords:** depression symptoms, health literacy, self-efficacy, social support, traditional Chinese medicine health literacy

## Abstract

**Background:**

Postpartum depressive symptoms are common and can adversely affect maternal and infant health. Traditional Chinese medicine health literacy may play an important role in promoting physical and mental recovery during the puerperium. However, evidence regarding the association between traditional Chinese medicine health literacy and postpartum depressive symptoms remains limited, and the underlying mediating mechanisms are unclear.

**Objective:**

To explore the relationship between Traditional Chinese Medicine health literacy and depressive symptoms in postpartum women, and to examine the sequential mediating role of social support and self-efficacy.

**Methods:**

Using convenience sampling, this cross-sectional study was conducted in Chengdu, China from December 2025 to April 2026. A total of 501 postpartum women completed measures of traditional Chinese medicine health literacy, social support, self-efficacy, and depressive symptoms. After adjusting for covariates, sequential mediation was examined by PROCESS 4.1 Model 6. Bias-corrected bootstrap (5,000 resamples) was used to generate 95% confidence intervals; indirect effects were deemed significant if the interval did not include zero. All tests were two-tailed with *α* = 0.05.

**Results:**

The scores of traditional Chinese medicine health literacy, postpartum social support, self-efficacy, and depressive symptoms were (118.21 ± 14.56), (47.72 ± 11.41), (29.22 ± 6.44), and (7.16 ± 4.99), respectively. Depressive symptoms were significantly negatively correlated with traditional Chinese medicine health literacy, social support, and self-efficacy (all *p* < 0.001). Traditional Chinese medicine health literacy exerted a direct negative effect on depressive symptoms (*β* = −0.206, 95% *CI*: −0.299 to −0.112). In addition, three significant indirect pathways were identified: the independent mediating pathway through social support (*β* = −0.102, 95% *CI*: −0.167 to −0.050), the independent mediating pathway through self-efficacy (*β* = −0.102, 95% *CI*: −0.148 to −0.059), and the sequential mediating pathway from social support to self-efficacy (*β* = −0.033, 95% *CI*: −0.053 to −0.018). The total indirect effect accounted for 53.6% of the total effect.

**Conclusion:**

Intervention strategies designed to improve postpartum women’s traditional Chinese medicine health literacy, strengthen social support and boost self-efficacy may aid the prevention and control of postpartum depressive symptoms. The actual effectiveness of relevant intervention programs needs to be further verified in subsequent longitudinal studies and intervention trials.

## 1 Introduction

Postpartum depression is a widely prevalent mental disorder that may affect mood and physical health, posing a serious challenge to postpartum women. A meta-analysis (1) involving 291 studies across 56 countries with 296,284 mothers reported a global pooled prevalence of postpartum depression of 17.7%, with substantial cross-country variation—3% in Singapore (2), an estimated 22.14% in China (3), and as high as 38% in Chile (1). Assessing the prevalence of depressive symptoms and exploring their underlying mechanisms is therefore crucial for protecting postpartum women from the harmful effects of depression (1, 2). While most research on depressive symptoms has focused on the broader postpartum period, studies specifically addressing the puerperium (the first 42 days postpartum) remain limited (4). In general postpartum populations, several correlates have been identified with postpartum depressive symptoms, including health literacy (5, 6), age, educational level, medical payment mode, planned pregnancy status and other factors (7). Notably, the traditional practice of “sitting the month” (Zuo Yuezi) during the puerperium is closely intertwined with Traditional Chinese Medicine (TCM). The majority of Chinese women adhere to this postpartum custom, which includes TCM dietary regimens, herbal remedies, activity restrictions, and emotional regulation practices (8). The effective application of TCM practices depends on women‘s ability to access, understand, evaluate, and utilize TCM-related health information—a capability defined as TCM health literacy (9), an extension of general health literacy within the TCM cultural context. However, empirical research exploring the correlation between TCM health literacy and depressive symptoms among postpartum women remains scarce, and the potential mediating mechanisms behind their association have not been clarified. Systematically sorting out correlations among various mediators can provide a theoretical basis for safeguarding the mental health of puerperal women.

### 1.1 Literature review and research hypothesis

#### 1.1.1 TCM health literacy and its direct association

Health literacy refers to individuals’ knowledge, attitudes and behavioral capacities to identify, manage and prevent diseases (10). Individuals with high health literacy are better at identifying, coping with, and preventing common diseases, and exhibit stronger willingness and capacity to seek professional assistance (11). Previous empirical studies have confirmed that health literacy is negatively correlated with postpartum depressive symptoms (12). As a context-specific derivative of health literacy, TCM health literacy develops under the influence of traditional Chinese medicine (13). TCM has long emphasized emotion regulation for puerperal women and been deeply integrated into daily postpartum confinement care for Chinese mothers (14). TCM health literacy is presumed to correlate with postpartum depressive symptoms as well. Accordingly, the following hypothesis is proposed: H1: TCM health literacy is negatively correlated with puerperal depressive symptoms.

#### 1.1.2 Social support and its mediating effect

Postpartum social support includes emotional, material, appraisal-related and informational support that parturient women receive from their surroundings (15). It serves as a critical protective factor for perinatal mental health (16). Meta-analytic evidence shows that inadequate social support is one of the major risk factors for postpartum depressive symptoms (17). Sustained low perceived social support after childbirth can worsen women’s depressive symptoms at 6 months postpartum (6). Traditional Chinese medicine emphasizes emotional care. It advocates physical and mental health maintenance and early prevention of mental disorders via emotion regulation, appropriate work-rest balance, reasonable diet and regular daily routines (18). Based on social support theory (19), women with higher TCM health literacy can articulate their physical and mental rehabilitation needs more clearly, They can reduce family disagreements over postpartum care and gain access to more social-support resources. Based on the above theoretical inferences, social support may act as a transmitting role between the two variables. Therefore, this study proposes the following hypothesis: H2: Social support mediates the association between TCM health literacy and postpartum depressive symptoms.

#### 1.1.3 Self-efficacy and its mediating effect

Self-efficacy refers to individuals’ subjective belief in their ability to cope with stress and achieve physical and mental recovery (20). It is an important cognitive factor affecting postpartum depressive symptoms (21). According to the Knowledge-Attitude-Practice theory (22), individual health cognition can sequentially shape health attitudes and psychological states. TCM health literacy reflects parturient women’s ability to understand and apply local knowledge of postpartum physical-constitution rehabilitation, daily diet regulation and emotional care (9). Women with higher TCM health literacy may gain clearer cognition and higher acceptance of postpartum rehabilitation approaches. They may further build positive beliefs for self-recovery and improve self-efficacy (6). Higher self-efficacy can reduce postpartum depressive symptoms (23). Taken together, these lines of evidence suggest certain associations among these variables. Self-efficacy may serve as a transmitting role between the two variables. Therefore, this study proposes the following hypothesis: H3: Self-efficacy mediates the association between TCM health literacy and postpartum depressive symptoms.

#### 1.1.4 The sequential mediating role of social support and self-efficacy

Existing studies have confirmed that social support is significantly positively correlated with self-efficacy, and both are negatively associated with postpartum depressive symptoms (24). According to the Capability-Opportunity-Motivation-Behaviour (COM-B) model (25), TCM health literacy represents the cognitive capability for puerperal women to conduct postpartum self-care (13); family social support refers to external social opportunities available to individuals, and self-efficacy serves as the internal motivational component driving women’s adaptation to postpartum life. The interaction of these three factors jointly promotes the occurrence of health-related behaviors (26). previous studies (11) indicated that puerperal women with higher general health literacy tend to have better social support, Theoretically, adequate social support can strengthen individuals’ internal efficacy beliefs and promote health-promoting behaviors (27). Based on the COM-B model, we propose the following hypothesis: H4: Social support and self-efficacy exert a sequential mediating effect on the association between TCM health literacy and postpartum depressive symptoms.

## 2 Method

### 2.1 Study population and sampling methods

Public data from the Chengdu Municipal Health Commission show that by the end of 2025, Chengdu had 70 tertiary Grade-A hospitals. However, no public statistics list the exact number of hospitals equipped with obstetric departments and 42-day postpartum re-examination clinics. Based on practical accessibility and field-implementation feasibility, we selected two tertiary Grade-A hospitals in Chengdu’s main urban area by convenience sampling. The recruitment period ran from December 2025 to April 2026. Both hospitals are located within the same administrative district. One was a university-affiliated general hospital with approximately 600 annual deliveries. The other was a municipal maternal and child health hospital with approximately 1,400 annual deliveries. Participants attending these hospitals came from multiple communities across the main urban area. Participants were also recruited using convenience sampling. Trained researchers conducted recruitment on fixed weekdays during regular clinic hours: Mondays and Tuesdays at the municipal maternal and child health hospital, and Wednesdays to Fridays at the affiliated general hospital. All eligible women who came for the 42-day postpartum check-up on these days were invited to take part in the study. Recruitment continued until the target sample size was achieved.

### 2.2 Sample size calculation

The required sample size was estimated using G*Power 3.1.9.7 software (28). The “Fixed model, *R*^2^ increase” mode for linear multiple regression was employed. The parameters were set as follows: *α* = 0.05 (two-tailed), statistical power (1 − *β*) = 0.90, and effect size *f^2^* = 0.065, which was derived from an identical sequential mediation model structure (29). A total of 22 predictors were specified in the model. The analysis indicated that a minimum sample size of 434 participants was required. Accounting for a 10% invalid response rate, the study planned to recruit at least 483 participants.

### 2.3 Inclusion and exclusion criteria

Inclusion criteria included: (1) Postpartum women who attended the routine 42-day postpartum re-examination at the hospital; (2) Aged 20 years or older; (3) Conscious and clear-minded, proficient in smartphone operation, and capable of independently completing the online questionnaire; (4) Voluntarily participating in this study.

Exclusion criteria included: (1) women with unstable and uncontrolled severe gestational or postpartum complications (such as severe preeclampsia, severe puerperal infection, postpartum hemorrhage); (2) Individuals with a confirmed history of mental illness, cognitive impairment, or consciousness disturbance, or those taking antipsychotic or antidepressant medications and unable to cooperate with the survey; (3) Participants whose newborns had severe congenital diseases or were admitted to the Neonatal Intensive Care Unit (NICU) and remained undischarged.

Questionnaire elimination criteria included: (1) Questionnaires with obvious regular-response patterns or a completion time of less than 5 min; (2) Participants who voluntarily withdrew from the study during the investigation.

### 2.4 Data collection process

The research team contacted the director of the obstetrics department, explained the purpose, significance, content, and questionnaire requirements of this study, and obtained their consent. Data were collected via the Wenjuanxing online platform, a professional survey tool widely used in Chinese academic research. The platform allows all questionnaire items to be set as mandatory, preventing submission if any item is missing; all data were encrypted and anonymized to ensure validity and ethical compliance. Eligible postpartum women were screened according to the inclusion and exclusion criteria and were invited to participate after providing informed consent. A pilot test indicated that it required at least 5 min to read and complete all items carefully; therefore, questionnaires completed in less than 5 min were considered invalid and excluded. This study was approved by the Ethics Committee of the Affiliated Hospital of Chengdu University (Approval No: PJ2025-137-01), and participants could withdraw voluntarily at any time without penalty. During the survey period, a total of 520 online questionnaires were distributed. Among them, 14 women declined to participate; 5 invalid questionnaires were excluded (including those with stereotyped response patterns and insufficient completion time), yielding 501 valid questionnaires, with an effective response rate of 96.35%, which ultimately met the predetermined sample size requirement.

### 2.5 Measurement tools

#### 2.5.1 Sociodemographic and obstetric information form

Designed by the researchers based on the study objectives, the questionnaire included items on age, occupation, education level, place of residence, number of antenatal education sessions, type of health insurance coverage, monthly household income per capita, planned pregnancy, mode of delivery, breastfeeding method and recruiting hospitals.

#### 2.5.2 Traditional Chinese medicine health literacy scale (TCM-HL-S)

This study adopted the scale developed by Yao (9) for assessment, which has been applied among puerperal women. The scale consists of 28 items covering three dimensions: basic knowledge, healthy lifestyle, and basic skills. Participants rated items on a 5-point Likert scale (1 = totally unaware to 5 = fully aware), with total scores ranging from 28 to 140; higher scores indicate higher levels of health literacy. The scale exhibits good reliability and validity. Its overall Cronbach’s *α* coefficient is 0.950, and the split-half reliability is 0.957. Developed via two rounds of Delphi consultations, the item-level content validity index (I-CVI) ranged from 0.800 to 1.000, and the scale-level average content validity index (S-CVI/Ave) was 0.960. Exploratory factor analysis extracted three common factors, accounting for a cumulative variance contribution of 61.173%. In the present study, the Cronbach’s α coefficient for the total scale was 0.918; This study Kaiser–Meyer–Olkin (KMO) value was 0.913, and Bartlett’s test of sphericity was statistically significant (*χ^2^* = 6594.184, df = 378, *p* < 0.001). These findings suggested good internal consistency of the scale in our study population and that the dataset was suitable for factor analysis, supporting its applicability for participants in this study.

#### 2.5.3 Postpartum social support scale (PSSS)

Developed by Lu and Zheng (15), this scale is used to assess the level of social support among postpartum women. It consists of 20 items across four dimensions: emotional support, material support, informational support, and appraisal support. Each item is rated on a 4-point Likert scale ranging from 0 (none) to 3 (a lot). Total scores range from 0 to 60, with higher scores indicating higher levels of social support. The content validity index was 0.90, and the overall Cronbach’s *α* coefficient was 0.89. This scale has been widely used among Chinese postpartum women. In this study, the Cronbach’s *α* coefficient was 0.968.

#### 2.5.4 General self-efficacy scale (GSES)

Developed by Schwarze and sinicized by Wang et al. (30), this scale was used to assess the overall confidence of postpartum women in coping with stress. It consists of 10 items, each rated on a 4-point Likert scale ranging from 1 (not at all true) to 4 (exactly true). Total scores range from 10 to 40, with higher scores indicating higher levels of general self-efficacy. In the original development study, the overall Cronbach’s *α* coefficient was 0.80, and the test–retest reliability was *r* = 0.83. The first eigenvalue was 5.226, accounting for 52.256% of the total variance. This scale has been widely used among puerperal women. In this study, the Cronbach’s *α* coefficient was 0.944.

#### 2.5.5 Edinburgh postnatal depression scale (EPDS)

In this study, the Chinese version of the Edinburgh Postnatal Depression Scale (EPDS) culturally adapted and revised by Yang (31). The scale consists of 10 items across three dimensions: anhedonia (Items 1–2), anxiety (Items 3–6), and depressive mood (Items 7–10). A 4-point Likert scale is applied (0 = not at all, 3 = most of the time), with Items 1 and 2 reverse-scored. Total scores range from 0 to 30, with higher scores indicating more severe depressive symptoms. A total score of ≥10 (32) was used as the cutoff for screening probable postpartum depressive symptoms. The three-factor structure of the scale has been validated in Chinese perinatal populations, with confirmatory factor analysis supporting its three-factor structure (CFI = 0.990, TLI = 0.978, RMSEA = 0.020, SRMR = 0.022); reliability was good (Cronbach‘s *α*: 0.81–0.85 for the total scale, 0.70–0.81 for subscales). In the present study, the Cronbach’s α coefficient of the total scale was 0.835.

### 2.6 Data analysis

Data were entered using Excel 2010 and analyzed with *SPSS* 23. The intraclass correlation coefficient (ICC) was calculated to test hospital clustering effects. Normality of the data was assessed using the *Q-Q* plots, and criteria of skewness <2 and kurtosis <7. Normally distributed continuous variables were described as mean ±standard deviation (*x- ± s*). while non-normally distributed continuous variables were presented as median (interquartile range). For normally-distributed data, Levene’s test was performed to assess homogeneity of variance for group comparisons, independent-samples *t*-tests were used for two-group comparisons and one-way analysis of variance (ANOVA) for multi-group comparisons. Welch-corrected tests would be adopted in cases of unequal variance. Non-normally distributed data were analyzed by nonparametric tests: the Mann–Whitney U test for two groups and the Kruskal–Wallis *H* test for multiple groups. Categorical variables were described with frequencies and percentages; inter-group comparisons were performed using the chi-square test or Fisher’s exact test. Common method bias was tested using Harman‘s single-factor test. Correlations among variables were analyzed using Pearson’s correlation. The sequential mediation effect was tested using Model 6 of Hayes ‘PROCESS macro for SPSS (version 4.1) for SPSS, with bootstrapping resampled 5,000 times. Bias-corrected 95% confidence intervals (CIs) were calculated; an indirect effect was considered statistically significant if the CI excluded zero. All statistical tests adopted a two-tailed significance level of *α* = 0.05.

## 3 Results

### 3.1 Test of ICC clustering effect

A two-level null linear mixed model was built to test hospital-based clustering from dual-site sampling. Within-hospital residual variance = 24.783 (Wald *Z* = 15.796, *p* < 0.001, 95% *CI*: 21.891 to 28.058), between-hospital intercept variance = 0.145 (Wald *Z* = 0.413, *p* = 0.680, 95% CI: 0.001 to 16.691). The intraclass correlation coefficient (ICC) was 0.006 (< 0.05) (33), showing negligible hospital-level clustering. Traditional mediation analysis was adopted for follow-up tests.

### 3.2 Normality test and common method bias

Normality analysis showed the absolute skewness values were less than 2 and absolute kurtosis values were below 7 (34) for health literacy, social support, self-efficacy and depressive symptom scores, indicating approximate normal distribution of these data. Since questionnaires were adopted in this study, Harman’s single-factor test was applied to assess common method bias. The results revealed that 11 factors had eigenvalues greater than 1, and the first common factor accounted for 30.987% of the total variance, which is below the recommended threshold of 40% (35). This indicates that common method bias is not a significant concern in this study.

### 3.3 Scores of TCM health literacy, social support, self-efficacy, and depressive symptoms among postpartum women

The total scores for TCM health literacy (TCM-HL), social support (SS), self-efficacy (SE), and depressive symptoms (DS) in this group of postpartum women were (118.21 ± 14.56), (47.72 ± 11.41), (29.22 ± 6.44), and (7.16 ± 4.99), respectively. The scores for each dimension are presented in Table 1. Using the clinically validated cutoff score of ≥10 on the EPDS (32), we identified the prevalence of postpartum depressive symptoms as 27.5% (95% CI: 23.6–31.5%).

**Table 1:** Scores of TCM-HL, SS, SE and DS (*N* = 501).

Item	Number of items	Score range	Score (*M ± SD*)	Item score (*M ± SD*)
**TCM-HL**	28	28 ~ 140	118.21 ± 14.56	4.22 ± 0.52
Knowledge	13	13 ~ 65	55.90 ± 7.36	4.30 ± 0.57
Lifestyle	8	8 ~ 40	35.08 ± 4.74	4.38 ± 0.59
Skills	7	7 ~ 35	27.23 ± 5.33	3.89 ± 0.76
**SS**	20	0 ~ 60	47.72 ± 11.41	2.39 ± 0.57
Emotional support	5	0 ~ 15	12.38 ± 2.78	2.48 ± 0.56
Material support	5	0 ~ 15	12.04 ± 3.04	2.41 ± 0.61
Informational support	5	0 ~ 15	11.04 ± 3.56	2.21 ± 0.71
Appraisal support	5	0 ~ 15	12.26 ± 3.08	2.45 ± 0.61
**SE**	10	10 ~ 40	29.22 ± 6.44	2.92 ± 0.64
**DS**	10	0 ~ 30	7.16 ± 4.99	0.72 ± 0.50
Anhedonia	2	0 ~ 6	1.06 ± 1.24	0.53 ± 0.62
Anxiety	4	0 ~ 12	3.59 ± 2.63	0.90 ± 0.66
Depressed mood	4	0 ~ 12	2.51 ± 2.40	0.63 ± 0.60

TCM-HL, Traditional Chinese Medicine Health Literacy; SS, Social Support; SE, Self-efficacy; DS, Depressive symptoms. M, mean; SD, standard deviation. Bold rows indicate main-scale scores, and indented rows indicate subscale or dimension scores.

### 3.4 Demographic characteristics and univariate analysis

Most participants were aged 26–30 years (55.1%); the majority held college or bachelor’s degrees (74.1%); The “other” occupation category had the highest proportion at 39.9%; 89.4% were permanent urban residents, 52.7% did not attend antenatal education sessions; 83.0% were covered by urban medical insurance; 83.2% had planned pregnancies.;43.3% had a monthly household per capita income of 5,000–7999 CNY, and 51.7% adopted exclusive breastfeeding. The results of univariate analysis showed that there were statistically significant differences (*p* < 0.05) in depressive symptom scores among postpartum women with different frequencies of antenatal education sessions, type of health insurance coverage, planned pregnancy status, and breastfeeding methods (see Table 2).

**Table 2:** Sociodemographic and obstetric characteristics of postpartum women (*N* = 501).

Characteristic	*n* (%)	Depressive symptoms	Statistic	*P*
Age			*F* = 0.681	0.564
≤25 years	54(10.8)	7.20 ± 5.04		
26–30 years	276(55.1)	7.05 ± 5.04		
31–35 years	138(27.5)	7.57 ± 5.12		
≥36 years	33(6.6)	6.30 ± 3.66		
Education level			*F* = 0.994	0.395
Middle school or below	50(9.9)	6.34 ± 4.60		
Technical secondary school or high school	53(10.6)	7.87 ± 4.24		
College or bachelor’s degree	371(74.1)	7.22 ± 5.18		
Master’s degree or above	27(5.4)	6.48 ± 4.12		
Occupation			*F* = 1.965	0.099
Public sector employee	40(8)	7.15 ± 4.48		
Healthcare worker	67(13.4)	6.96 ± 5.27		
Full-time mother	186(37.1)	7.94 ± 5.35		
Farmer	8(1.6)	6.63 ± 5.18		
Other	200(39.9)	6.54 ± 4.56		
Residence			*t* = −0.271	0.786
Urban	448(89.4)	7.14 ± 5.00		
Rural	53(10.6)	7.34 ± 4.89		
Number of antenatal education sessions			*F* = 5.202	**0.006**
0	264(52.7)	6.49 ± 4.98		
1 ~ 3	166(33.1)	7.92 ± 4.61		
≥ 4	71(14.2)	7.92 ± 5.55		
Monthly household income per capita			*F* = 1.230	0.298
<2000 CNY	14(2.8)	7.5 ± 5.13		
2000–4,999 CNY	155(30.9)	7.48 ± 4.58		
5,000–7,999 CNY	217(43.3)	6.68 ± 5.05		
≥8,000 CNY	115(23)	7.61 ± 5.35		
Type of health insurance coverage			*F* = 10.619	**0.001**
Urban medical insurance	416(83)	7.05 ± 4.85		
New Rural Cooperative Medical System	60(12)	6.22 ± 4.99		
Other	25(5)	11.4 ± 5.39		
Mode of delivery			*t* = 0.645	0.519
Vaginal delivery	263(52.5)	7.30 ± 4.89		
Cesarean section	238(47.5)	7.01 ± 5.09		
Planned pregnancy			*t* = −2.003	**0.046**
Yes	417(83.2)	6.96 ± 4.97		
No	84(16.8)	8.15 ± 4.98		
Feeding method			*F* = 5.189	**0.006**
Exclusive breastfeeding	259(51.7)	6.48 ± 4.70		
Mixed feeding	189(37.7)	7.85 ± 5.16		
Formula feeding	53(10.6)	8.08 ± 5.34		
Recruiting hospitals			*t* = 1.511	0.122
Municipal Maternal and Child Health Hospital	199(39.7)	7.44 ± 4.97		
Affiliated Hospital	302(60.3)	6.74 ± 4.99		

Measurement data were presented as mean ± standard deviation, and count data were presented as *n* (%). Levene’s test indicated homogeneity of variance (*p* > 0.05). Independent-samples *t*-test was used for two-group comparisons, and one-way ANOVA was used for multi-group comparisons. Values in bold indicate statistically significant differences (*p* < 0.05).

### 3.5 Correlation analysis

Correlation results are detailed in Table 3. DS was significantly negatively correlated with TCM-HL, SS and SE. TCM-HL was significantly positively correlated with SS and SE, and SS was significantly positively correlated with SE. These findings confirm notable correlations between core variables and support further analysis of indirect effects (see Table 3).

**Table 3:** Correlations among TCM-HL, SS, SE and DS (*N* = 501).

Variables	SE	TCM-HL	SS	DS
SE	1			
TCM-HL	0.551^**^	1		
SS	0.497 ^**^	0.520^**^	1	
DS	−0.438^**^	−0.452^**^	−0.408^**^	1

TCM-HL, Traditional Chinese Medicine Health Literacy; SE, self-efficacy; SS, social support; DS, depressive symptoms. **p < 0.01.

### 3.6 Sequential mediation model

In this study, TCM-HL and DS were specified as the focal variables, with SS and SE examined as variables involved in a sequential indirect association pattern. PROCESS Model 6 estimated four separate regression equations: Model 1 estimated the total effect of TCM-HL on DS; Model 2 regressed SS on TCM-HL; Model 3 regressed SE on TCM-HL and SS; and Model 4 regressed DS on TCM-HL, SS, and SE. All models were adjusted for age, educational level, monthly household income per capita, and variables with statistical significance in the univariate analysis, including number of antenatal education sessions, type of health insurance coverage, planned pregnancy, and feeding method, to control for potential confounding factors. The regression results showed that TCM-HL significantly predicted SS, TCM-HL and SS significantly predicted SE, and TCM-HL, SS, and SE significantly predicted DS in the final model. These models explained 33.2% of the variance in SS, 41.3% of the variance in SE, and 36.1% of the variance in DS, respectively. Detailed regression results are presented in Table 4, and the adjusted standardized path coefficients are shown in Figure 1. Bootstrap analysis with 5,000 resamples yielded a standardized total effect of −0.444, and a standardized direct effect of −0.206 for TCM-HL on DS. The standardized total indirect effect was −0.238, accounting for 53.6% of the total effect. Three distinct significant standardized indirect pathways were identified: TCM-HL → SS → DS (*β* = −0.102, 95% *CI*: −0.167 to −0.050), TCM-HL → SE → DS (*β* = −0.102, 95% *CI*: −0.148 to −0.059), and the sequential pathway TCM-HL → SS → SE → DS (*β* = −0.033, 95% *CI*: −0.053 to −0.018). All indirect effects reached statistical significance, as their confidence intervals excluded zero. Full estimates of standardized direct and indirect effects are listed in Table 5. Collectively, these cross-sectional findings support Hypotheses H1–H4 at the correlational level. Owing to the cross-sectional study design, causal mediation inferences cannot be drawn from the results.

**Table 4:** Regression results for the sequential mediation model examining the association between TCM-HL and DS through SS and SE (PROCESS Model 6, *N* = 501).

Model	Dependent variable	Predictor	*B*	Std error	*P*	95% *CI*	*β*
Model 1	DS	TCM-HL	−0.152	0.014	<0.001	(−0.179, −0.125)	−0.444
Model 2	SS	TCM-HL	0.372	0.030	<0.001	(0.312, 0.432)	0.475
Model 3	SE	TCM-HL	0.182	0.018	<0.001	(0.145, 0.218)	0.411
		SS	0.159	0.024	<0.001	(0.112, 0.207)	0.282
Model 4	DS	TCM-HL	−0.07	0.016	<0.001	(−0.103, −0.038)	−0.206
		SS	−0.094	0.02	<0.001	(−0.134, −0.054)	−0.215
		SE	−0.193	0.037	<0.001	(−0.265, −0.121)	−0.249

B, unstandardized regression coefficients; std error, standard error; 95% CI, 95% confidence interval; β, standardized coefficients. All models controlled for age, educational level, monthly household income per capita, number of antenatal education sessions, type of health insurance coverage, planned pregnancy, and feeding method. TCM-HL, Traditional Chinese Medicine Health Literacy; SE, self-efficacy; SS, social support; DS, depressive symptoms.

**Figure 1:**
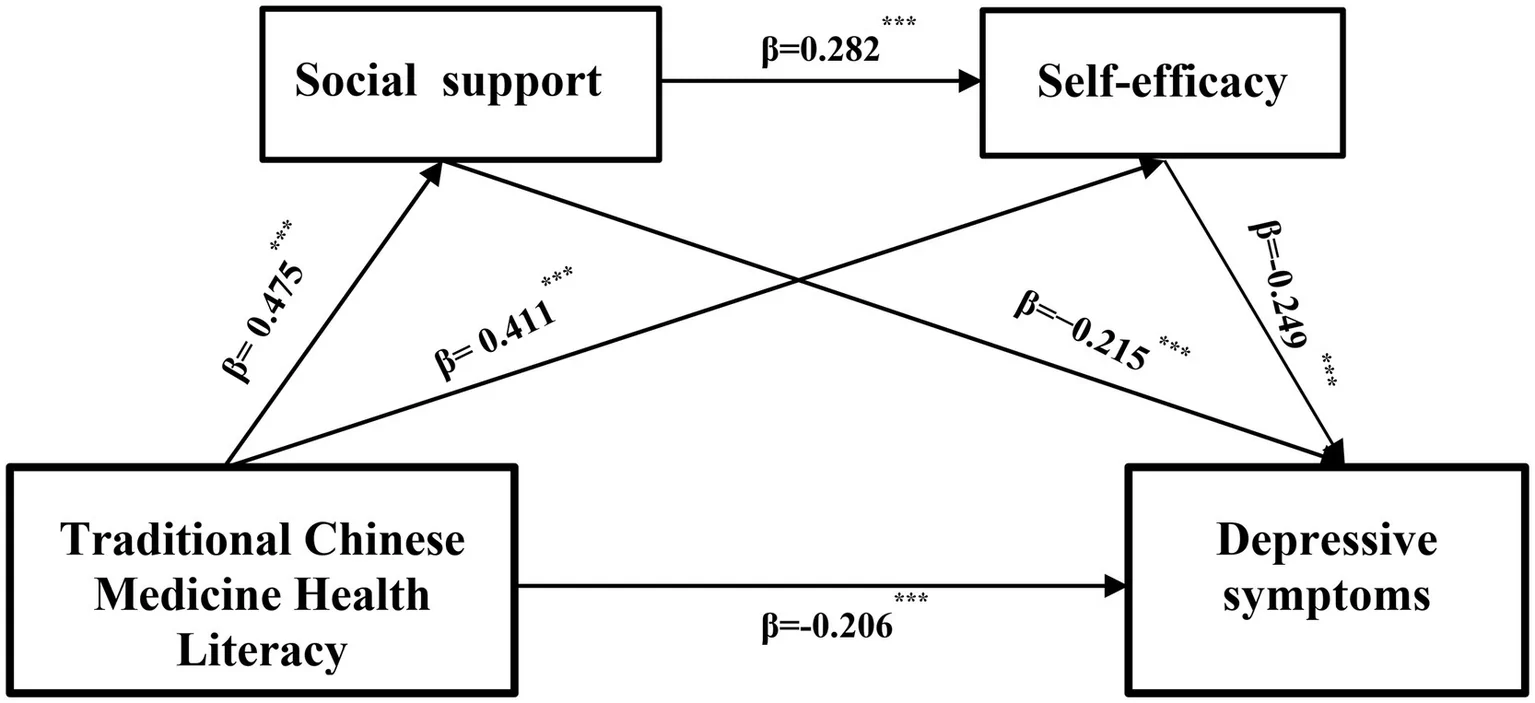
Sequential mediation model of the association between Traditional Chinese Medicine health literacy and depressive symptoms through social support and self-efficacy. Values of *β* represent standardized path coefficients. The model was adjusted for age, educational level, monthly household income per capita, number of antenatal education sessions, type of health insurance coverage, planned pregnancy, and feeding method. ***p < 0.001.

**Table 5:** Total, direct, and indirect effects of TCM-HL on DS through SS and SE (*N* = 501).

Effect type	*β*	BootSE	Bootstrap 95%	Proportion of total effect (%)
Total effect	−0.444	0.041	(−0.524, −0.364)	100
Direct effect	−0.206	0.048	(−0.299, −0.112)	46.4
Total indirect effect	−0.238	0.035	(−0.311, −0.174)	53.6
TCM-HL → SS → DS	−0.102	0.029	(−0.167, −0.05)	23
TCM-HL → SE → DS	−0.102	0.023	(−0.148, −0.059)	23.1
TCM-HL → SS → SE → DS	−0.033	0.009	(−0.053, −0.018)	7.5

Comparison of total, direct and indirect effects of TCM-HL on DS, serially mediated by SS and SE. TCM-HL, Traditional Chinese Medicine Health Literacy; SS, Social Support; SE, Self-efficacy; DS, Depressive symptoms.; β, standardized coefficients; BootSE, Bootstrap-derived standard error. The indirect effect was statistically significant if the 95% bootstrap confidence interval did not include zero. The standardized coefficients in this table and Figure 1 are based on ordinary least-squares (OLS) regression using the full original sample, whereas the results in this table are derived from 5,000 bootstrap resamples. The minor difference of 0.001 between the two estimates reflects differences in computational logic, random sampling variation, and decimal-rounding rules across software, which is a normal occurrence in mediation analysis.

## 4 Discussion

### 4.1 Current status and cause analysis of variable scores in postpartum women

This study found that postpartum women had a total TCM-related health literacy score of 118.21 ± 14.56, higher than results from Yao (9). This difference may stem from participants’ educational levels and national promotion of TCM-based maternal-child health education (36). The skill dimension scored lowest, suggesting postpartum women possess sufficient theoretical knowledge but limited practical skills (37). Participants obtained a total social support score of 47.72 ± 11.41, comparable with earlier studies (15). The information support dimension had the lowest score, likely due to poor access to ongoing health information after hospital discharge (38). Their postpartum self-efficacy score was 29.22 ± 6.44, higher than prior reports (39). This could reflect the sample’s relatively high education and household economic status. Notably, the screening prevalence of postpartum depressive symptoms was 27.5%, comparable with earlier studies (32). Anxiety items showed high scores, which may partly relate to high urban living costs and extra financial burdens from childcare (40). These results indicate postpartum mental health screening should target not only depressive symptoms, but also early identification and assessment of anxiety.

### 4.2 Direct association between TCM health literacy and depressive symptoms

This study found a significant association between TCM-related health literacy and puerperal depressive symptoms. This result supported Hypothesis H1. It also agreed with previous findings on health literacy (27). Health promotion theory suggests that people with higher health literacy can acquire, understand and use health information more efficiently, They have better health awareness, This enables them to identify negative emotions in a timely manner (41). Most earlier studies examined the links between general health literacy and postpartum depression (37, 42). Different from previous studies, this study enriches the understanding of health literacy in the context of Chinese traditional Chinese medicine culture. TCM-based postpartum care is widely practiced and culturally recognized among Chinese postpartum women (43, 44). Therefore, TCM-related health literacy has unique research value for this population. This study provides a theoretical basis for implementing postpartum mental-health promotion work.

### 4.3 Discussion on indirect association

#### 4.3.1 The indirect effect of social support between TCM health literacy and depressive symptoms

This study revealed that social support exerted a significant mediating effect on the association between TCM-related health literacy and depressive symptoms among puerperal women. This finding supported Hypothesis H2. It was consistent with previous studies (37). Social support played an indirect role in the relationship between health literacy and depressive symptoms (45). According to social-support theory (19), women with higher TCM-related health literacy may access richer external resources. On the one hand, family members can share parenting stress. Women can obtain informational support by learning TCM-based postpartum care knowledge. They can also gain relevant emotional and appraisal support. On the other hand, women with higher TCM-related health literacy have clearer understanding of postpartum physical changes and postpartum rehabilitation approaches. They are more willing to express their physical and mental needs to family members and reduce psychological burden in childcare (16). Future studies may test whether intervention strategies targeting improved TCM-related health literacy and enhanced social support can alleviate depressive symptoms.

#### 4.3.2 The indirect effect of self-efficacy between TCM health literacy and depressive symptoms

Our findings suggested that self-efficacy may exert a mediating effect on the association between TCM-related health literacy and depressive symptoms. This result supported Hypothesis H3. It aligned with empirical findings reported (11). Regarding the mediating role of self-efficacy between health literacy and depressive symptoms. According to social cognitive theory (20), self-efficacy originates mainly from mastery experiences and vicarious experiences (observing others). Theoretically, adequate TCM-related health literacy is associated with puerperal women’s development of scientific postpartum-care perceptions and accumulation of positive experiences. It may further improve self-efficacy and help women actively manage their emotions. Nevertheless, such directional inferences need to be further tested in longitudinal studies.

#### 4.3.3 Social support and self-efficacy exert a sequential mediating effect between TCM health literacy and depressive symptoms

The sequential mediation model test showed that TCM-related health literacy could be associated with depressive symptoms among puerperal women through two independent mediating paths of social support and self-efficacy. It also verified Hypothesis H4,by the sequential pathway of “social support →self-efficacy”. The puerperium is a special period for women’s physical and psychological adjustment (46). It is often accompanied by physical debilitation and prominent parenting-related stress. Physical and mental recovery is related to health literacy (37, 42). According to social cognitive theory (20), this sequential pathway can be divided into three parts. First, puerperal women with good TCM-related health literacy can obtain professional knowledge, including TCM constitution regulation, postpartum diet guidance, emotion management, and home-based rehabilitation. With this knowledge, they can correctly recognize their physical and mental conditions and actively voice their healthcare needs. As a result, they tend to report higher perceived social support (37, 45). Second, adequate social support can buffer postpartum stress, improve psychological adjustment, and boost maternal parenting confidence as well as self-efficacy (24). Third, improved self-efficacy enables puerperal women to actively cope with diverse postpartum challenges, maintain favorable mental status, and is closely associated with lower levels of postpartum depressive symptoms (23, 47). Notably, this sequential pathway has also been validated in other populations (48, 49). Therefore, this study considers the combined roles of TCM-related health literacy, social support, and self-efficacy. It provides theoretical insights for future research on the influencing pathways of depressive symptoms among puerperal women. However, the full sequential chain is based on cross-sectional data. It represents only a theoretical interpretation. Longitudinal studies are needed for further validation.

## 5 Limitations

This study has several limitations. First, convenience sampling was adopted. Participants were recruited only from the 42-day postpartum follow-up clinics of two tertiary hospitals, with high-risk groups for postpartum depression excluded. Restrictions on online questionnaire completion and follow-up eligibility led to enrolled participants having generally better health status and higher digital literacy. The underrepresentation of women with scarce resources, limited digital access and high depression risk restricts the generalizable scope of findings. Second, all variables were measured by one-time online self-reported questionnaires. Although Harman’s single-factor test was performed, it has limited efficacy in controlling common method bias, and potential response biases cannot be fully ruled out. Third, the chained mediation model was constructed based on established theories to verify core pathways. Given the multifactorial nature of postpartum depression symptoms, this model cannot cover all underlying mechanisms, and alternative variable combinations and action pathways remain possible. Fourth, the cross-sectional design cannot clarify variable time sequences or confirm causal relationships. Mediation results only indicate statistical correlations, and reciprocal or reverse effects between variables may exist. Fifth, the TCM health literacy scale was developed and validated for Chinese populations. Its cross-cultural applicability lacks sufficient verification, so caution is needed when extrapolating present findings.

## 6 Conclusion

The results reveal that TCM health literacy has a direct correlation with postpartum depressive symptoms. It also forms multiple layers of indirect correlations through two independent pathways (social support and self-efficacy) and their sequential mediating pathway. Running through all correlational pathways, TCM health literacy highlights the practical significance of improving such literacy. Accordingly, designing integrated service schemes centered on optimizing social support and boosting self-efficacy can serve as a valuable reference for refining postpartum mental health interventions. These findings broaden the theoretical research perspective on correlational mechanisms of postpartum depressive symptoms, and offer practical entry points for developing targeted comprehensive intervention strategies.

## Data Availability

The original contributions presented in the study are included in the article/Supplementary material, further inquiries can be directed to the corresponding author.
