# Supplementary material for: Traditional Chinese medicine health literacy and depressive symptoms in postpartum women: the sequential mediating effects of social support and self-efficacy
Source: Front Public Health. 2026 Sep 16;14:1891379. doi: 10.3389/fpubh.2026.1891379 (PMC13625274; doi:10.3389/fpubh.2026.1891379)
Supplement: Supplementary file 1 [file Data_Sheet_1.PDF]

Supplementary Material

1 Normality test

Normality of the total scores of **TCM health literacy** and **Depressive Symptoms** was assessed by skewness, kurtosis

			Statistic	Std. Error
**Depressive symptom**	Mean		7.16	.223
	95% Confidence Interval for Mean	Lower Bound	6.73	
		Upper Bound	7.60	
	5% Trimmed Mean		6.90	
	Median		6.00	
	Variance		24.853	
	Std. Deviation		4.985	
	Minimum		0	
	Maximum		24	
	Range		24	
	Interquartile Range		7	
	Skewness		.761	.109
	Kurtosis		.112	.218
**TCM health literacy**	Mean		118.21	.651
	95% Confidence Interval for Mean	Lower Bound	116.93	
		Upper Bound	119.49	
	5% Trimmed Mean		119.09	
	Median		120.00	
	Variance		212.097	
	Std. Deviation		14.564	
	Minimum		45	
	Maximum		140	
	Range		95	
	Interquartile Range		18	
	Skewness		-1.090	.109
	Kurtosis		2.445	.218

Normal Q-Q Plot for total score of TCM health literacy

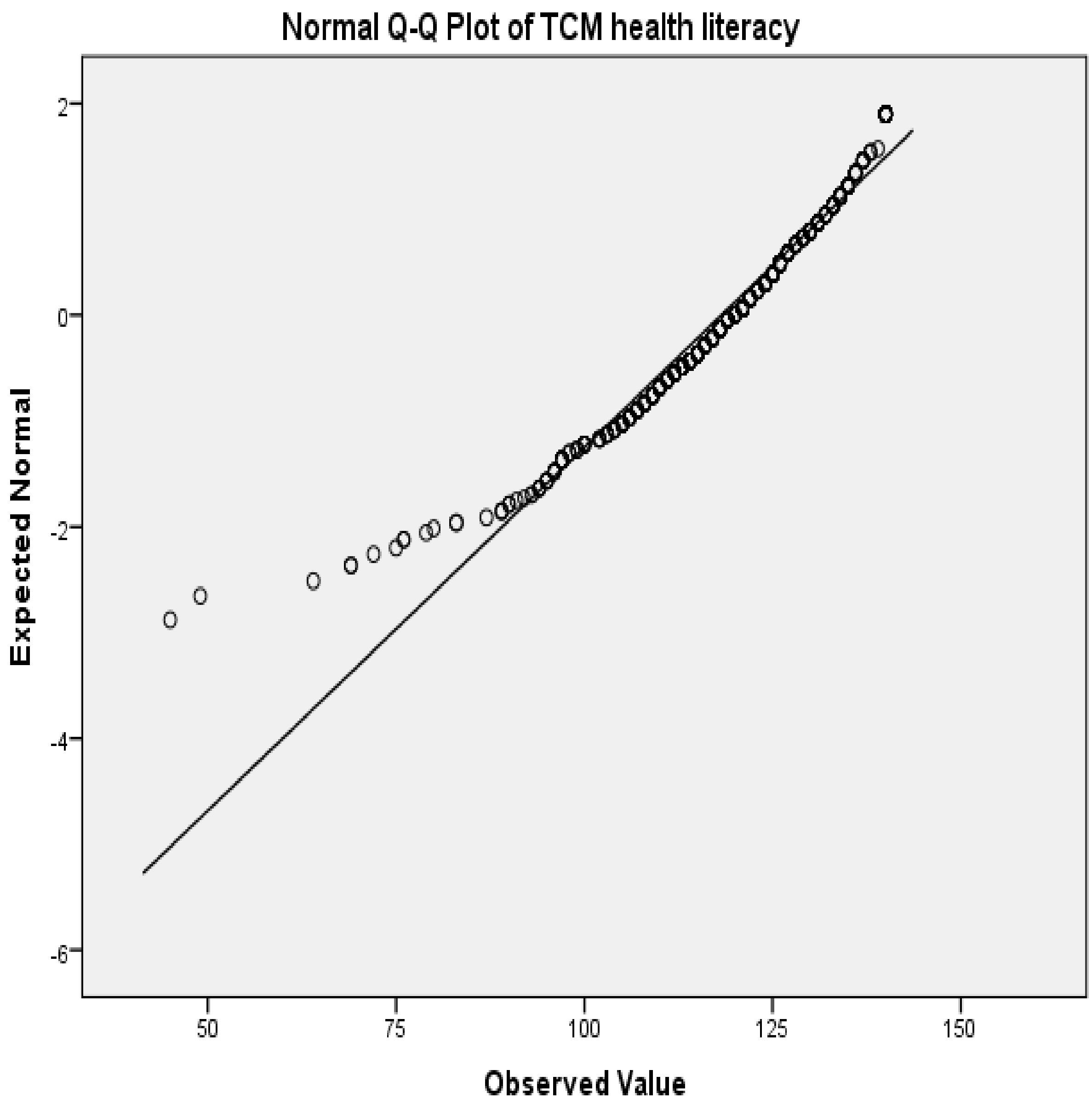

Normal Q-Q Plot for total score of depressive symptoms

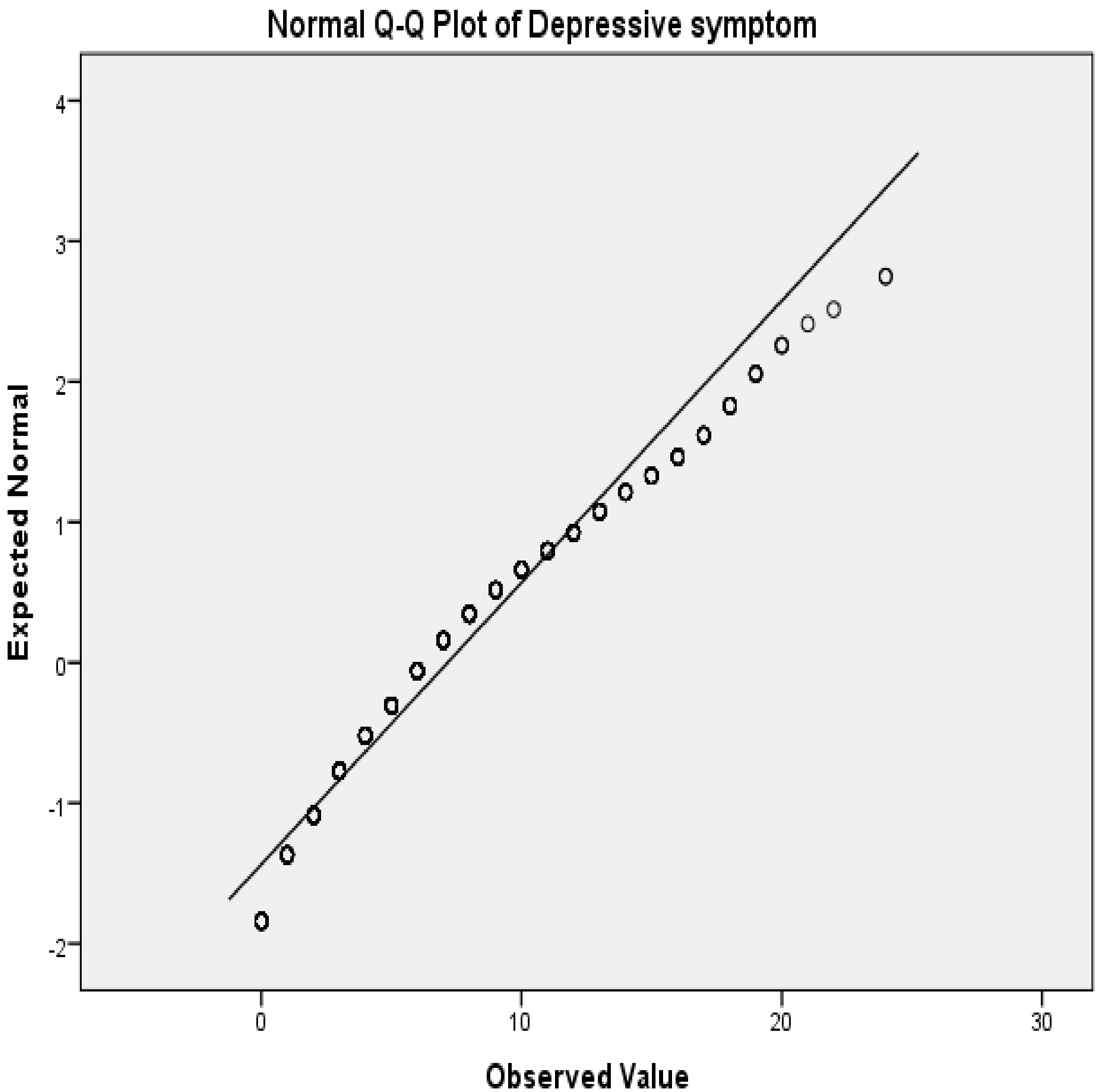

Normality of the total scores of social support and self-efficacy was assessed by skewness and kurtosis.

			Statistic	Std. Error
self-efficacy	Mean		29.59	.285
	95% Confidence Interval for Mean	Lower Bound	29.03	
		Upper Bound	30.15	
	5% Trimmed Mean		29.71	
	Median		30.00	
	Variance		40.598	
	Std. Deviation		6.372	
	Minimum		10	
	Maximum		40	
	Range		30	
	Interquartile Range		9	
	Skewness		-.095	.109
	Kurtosis		-.458	.218
social support	Mean		47.72	.510
	95% Confidence Interval for Mean	Lower Bound	46.72	
		Upper Bound	48.72	
	5% Trimmed Mean		48.63	
	Median		50.00	
	Variance		130.163	
	Std. Deviation		11.409	
	Minimum		8	
	Maximum		60	
	Range		52	
	Interquartile Range		18	
	Skewness		-.871	.109
	Kurtosis		.317	.218

Normal Q-Q Plot for total score of social support

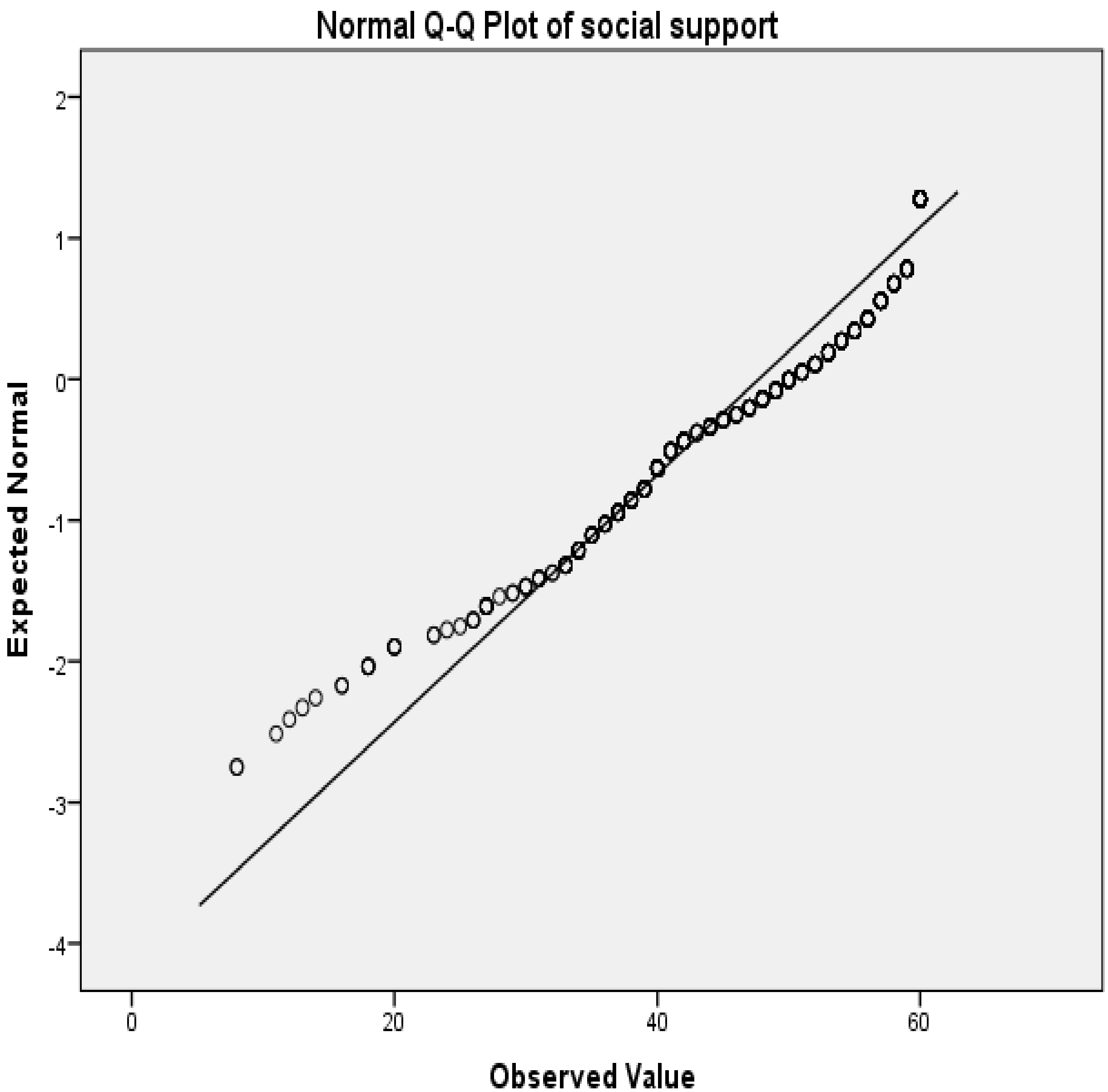

Normal Q-Q Plot for total score of self-efficacy

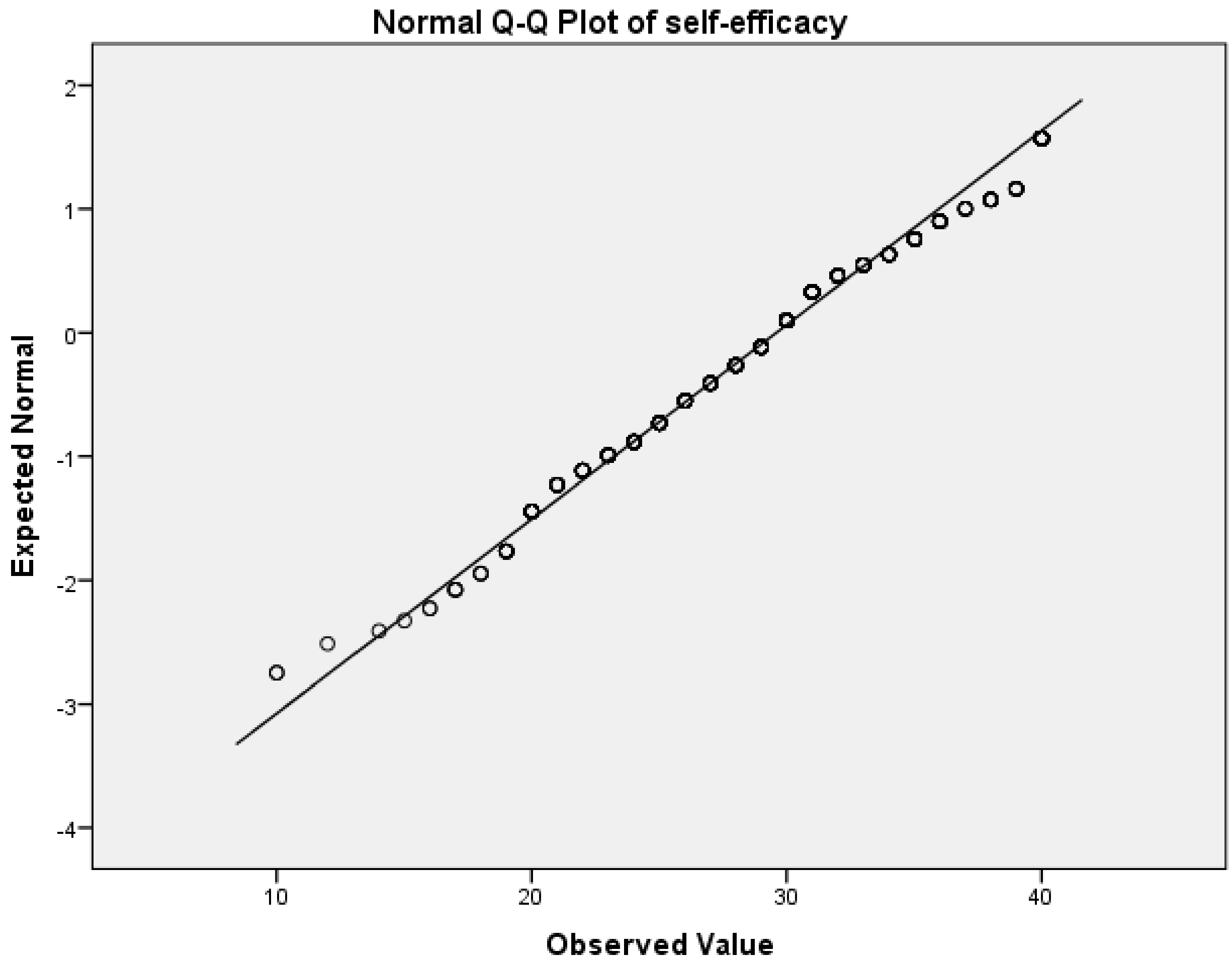

2 Harman's one-factor test

Total Variance Explained

Component	Initial Eigenvalues			Extraction Sums of Squared Loadings		
	Total	% of Variance	Cumulative %	Total	% of Variance	Cumulative %
1	21.133	31.078	31.078	21.133	31.078	31.078
2	5.770	8.486	39.563	5.770	8.486	39.563
3	4.675	6.874	46.438	4.675	6.874	46.438
4	3.334	4.903	51.341	3.334	4.903	51.341
5	1.952	2.870	54.211	1.952	2.870	54.211
6	1.764	2.595	56.806	1.764	2.595	56.806
7	1.663	2.445	59.251	1.663	2.445	59.251
8	1.465	2.154	61.405	1.465	2.154	61.405
9	1.102	1.620	63.025	1.102	1.620	63.025
10	1.059	1.558	64.583	1.059	1.558	64.583
11	1.052	1.547	66.129	1.052	1.547	66.129
12	.929	1.366	67.496			
13	.905	1.331	68.826			
14	.888	1.306	70.132			
15	.852	1.254	71.386			
16	.803	1.181	72.567			
17	.748	1.100	73.667			
18	.732	1.076	74.743			
19	.689	1.013	75.756			
20	.681	1.002	76.758			
21	.645	.948	77.706			
22	.644	.948	78.654			
23	.629	.925	79.579			
24	.592	.871	80.449			
25	.574	.845	81.294			
26	.560	.824	82.118			
27	.522	.768	82.885			
28	.511	.752	83.637			
29	.493	.726	84.363			
30	.469	.689	85.052			
31	.462	.679	85.731			
32	.442	.649	86.380			
33	.438	.644	87.025			
34	.414	.609	87.634			
35	.397	.584	88.218			
36	.390	.574	88.791			

37	.386	.568	89.360			
38	.364	.536	89.895			
39	.356	.524	90.419			
40	.352	.517	90.936			
41	.337	.495	91.432			
42	.326	.480	91.912			
43	.323	.474	92.386			
44	.318	.467	92.853			
45	.307	.452	93.305			
46	.296	.435	93.740			
47	.288	.424	94.164			
48	.269	.395	94.559			
49	.261	.383	94.942			
50	.247	.364	95.306			
51	.241	.354	95.660			
52	.232	.341	96.001			
53	.222	.326	96.327			
54	.216	.318	96.645			
55	.208	.306	96.951			
56	.204	.300	97.251			
57	.199	.292	97.543			
58	.189	.278	97.821			
59	.183	.269	98.090			
60	.174	.256	98.346			
61	.171	.252	98.597			
62	.161	.237	98.834			
63	.157	.230	99.065			
64	.145	.213	99.278			
65	.137	.201	99.479			
66	.125	.183	99.663			
67	.121	.178	99.840			
68	.109	.160	100.000			

Extraction Method: Principal Component Analysis.

Table 4 Variable coding

Independent variable	Coding
Number of antenatal education sessions	0 times = 1; 1–3 times = 2; ⩾4 times = 3
Payment method for hospitalization	Urban medical insurance = (0,0); New Rural Cooperative Medical System = (1,0); Other = (0,1)
Planned pregnancy	No = 0; Yes = 1
Feeding method	Exclusive breastfeeding = (0,0); Mixed feeding = (1,0); Formula feeding = (0,1)
social support	Raw value entered
self-efficacy	Raw value entered
TCM health literacy score	Raw value entered
